# Three-dimensional printed polylactic acid scaffold integrated with BMP-2 laden hydrogel for precise bone regeneration

**DOI:** 10.1186/s40824-021-00233-7

**Published:** 2021-10-27

**Authors:** Misun Cha, Yuan-Zhe Jin, Jin Wook Park, Kyung Mee Lee, Shi Huan Han, Byung Sun Choi, Jae Hyup Lee

**Affiliations:** 1 Biotechnology Institute, Medifab Co. LTD., 70, Dusan-ro, Doksan-dong, Geumcheon-gu, Seoul, 085-84 South Korea; 2 Department of Orthopedic Surgery, SMG-SNU Boramae Medical Center, 39 Boramae Gil, Dongjak-Gu, Seoul, 156-707 South Korea; 3 Department of Orthopedic Surgery, College of Medicine, Seoul National University, Seoul, 110-799 South Korea; 4 Spine Department, The First Hospital of Jilin University, Changchun, 130031 China; 5 Jilin Engineering Research Center for Spine and Spinal Cord Injury, Changchun, China; 6 Institute of Medical and Biological Engineering, Seoul National University Medical Research Center, Seoul, 110-799 South Korea

**Keywords:** 3D scaffold, 3D printing, Biogel, Critical bone defect, Controlled release, BMP-2, Misun Cha and Yuan-Zhe Jin contributed equally to this work and should both be considered as first authors.

## Abstract

**Background:**

Critical bone defects remain challenges for clinicians, which cannot heal spontaneously and require medical intervention. Following the development of three-dimensional (3D) printing technology is widely used in bone tissue engineering for its outstanding customizability. The 3D printed scaffolds were usually accompanied with growth factors, such as bone morphometric protein 2 (BMP-2), whose effects have been widely investigated on bone regeneration. We previously fabricated and investigated the effect of a polylactic acid (PLA) cage/Biogel scaffold as a carrier of BMP-2. In this study, we furtherly investigated the effect of another shape of PLA cage/Biogel scaffold as a carrier of BMP-2 in a rat calvaria defect model and an ectopic ossification (EO) model.

**Method:**

The PLA scaffold was printed with a basic commercial 3D printer, and the PLA scaffold was combined with gelatin and alginate-based Biogel and BMP-2 to induce bone regeneration. The experimental groups were divided into PLA scaffold, PLA scaffold with Biogel, PLA scaffold filled with BMP-2, and PLA scaffold with Biogel and BMP-2 and were tested both in vitro and in vivo. One-way ANOVA with Bonferroni post-hoc analysis was used to determine whether statistically significant difference exists between groups.

**Result:**

The in vitro results showed the cage/Biogel scaffold released BMP-2 with an initial burst release and followed by a sustained slow-release pattern. The released BMP-2 maintained its osteoinductivity for at least 14 days. The in vivo results showed the cage/Biogel/BMP-2 group had the highest bone regeneration in the rat calvarial defect model and EO model. Especially, the bone regenerated more regularly in the EO model at the implanted sites, which indicated the cage/Biogel had an outstanding ability to control the shape of regenerated bone.

**Conclusion:**

In conclusion, the 3D printed PLA cage/Biogel scaffold system was proved to be a proper carrier for BMP-2 that induced significant bone regeneration and induced bone formation following the designed shape.

**Supplementary Information:**

The online version contains supplementary material available at 10.1186/s40824-021-00233-7.

## Introduction

The critical bone defects that cannot heal spontaneously remain a major challenge for clinicians. In these cases, autografts are still considered as gold standard, but their usages are restricted because of their limited supply, complications at the donor site, variety in donor condition [1–3]. Following the development in three-dimensional (3D) printing technology, 3D printed customized bone substitutes have become an alternative choice of autograft for repairing bone defects [4]. 3D printing technique has unique advantages in customizing that can satisfy different shapes of bone defect of the patients, offering precise and customized treatment to the patients based on their CT data. In bone repairing, polylactic acid (PLA) is widely used for 3D printing tissue engineering scaffolds for its degradability, compatibility, and mechanical properties [5–8]. The process of bone regeneration is a series of biological events that involve adhesion of stem cells to the defect and inducing osteoblastic differentiation of the cells. Among the various osteoinductive growth factors, bone morphogenetic protein-2 (BMP-2) is the most widely used one in clinic, which promotes osteoblast differentiation and accelerates the repair of bone defects. As a water-soluble growth factor, the BMP-2 needs an appropriate carrier to maintain its concentration in the defect, and which mainly determined the effect of BMP-2 [9]. However, it is still a major challenge to find an optimal carrier of it, which could promote the bone regeneration and simultaneously guide the regenerated bone to locate at the proper location with a proper shape to avoid ectopic ossification (EO) that causes a series of complications such as overt dysfunction, soft-tissue loss, joint contractures, chronic pain.

Adsorption to collagen sponges or soaking of collagen sponges in BMP-2 are the most commonly used approaches to carrier design owing to the high binding capacities [10]. However, there are several disadvantages associated with the use of these naturally-derived polymers, including limited control over the mechanical properties, biodegradability, and batch-to-batch consistency [11]. In view of these limitations, synthetic polymers are beneficial because their mechanical properties, microstructure, and degradation rate can be predominantly controlled by their composition and fabrication technique [12]. However, synthetic polymers must be used to modify their surface with several active compounds, such as poly(L-lysine), heparin, or RGD peptides, or treatment with plasma or UV light is necessary to functionalize the polymers with BMP-2 [13, 14].

Previously, we have proved the 3D printed PLA scaffold combined with Biogel to be a proper carrier of rhBMP-2 and induced substantial bone regeneration in tibia defect [10].. In this study, we furtherly evaluated the effect of the scaffold as a carrier of the BMP-2 in rat calvaria defect model and rat back muscle model for a better understanding of its effect on inducing bone regeneration following designed shape. We hypothesized that Biogel play the important role maintains the biological activity of drug, releases it in a controlled manner at the surgical site, and prevents systemic diffusion of implanted drug. It was demonstrated as 3D printed cage/Biogel /BMP-2 system serve as an implant platform for precise bone tissue regeneration in the original defect area.

## Materials and methods

### Materials

Pure PLA filament was purchased from MakerBot (New York City, NY, USA). Alginate and gelatin-based Biogel was supplied by MediFab Co., Ltd. (Seoul, Korea). All remaining chemicals were purchased from Sigma-Aldrich (St. Louis, MO, USA).

### Cage design and fabrication

The cages were designed with a solid modeling software (SolidWorks®, Dassault Systèmes SolidWorks Corp.) and the data was saved as a stereolithography (.stl) file that could be directly imported into the 3D printer software. The cage was designed as a hollow cylinder with a height of 3 mm, diameter of 8 mm and the cavity has an inner height of 2 mm, inner diameter of 7 mm. Twelve rectangle holes with 1 mm edges were designed on the upside and downside of the cage for releasing BMP-2 from the cage. (Supplementary Figure 1) A basic and commercially available Desktop 3D printer (Replacator™2, Makerbot) was used in this study, and a cartridge is installed to supply the feedstock PLA filament (Makerbot, Co., Ltd., USA). The melted PLA filament (Makerbot, Co., Ltd) was extruded through a heated metal nozzle (205 °C) with 0.2 mm in diameter and horizontal and vertical movements. The extruded PLA was deposited onto a receiving station to form the desired scaffolds.

### Preparation of cages containing biogel-loaded with BMP-2

Following the manufacturer’s recommendation, the Biogel was warmed at 37 °C for 30 min and was mixed with BMP-2. After BMP-2 and the pre-warmed Biogel solution were uniformly mixed, the solution was injected into the cages through a rectangular hole. Then Biogel-loaded cages were gelated by immersion in casting buffer (Medifab Co. Ltd., Seoul, Korea) at 4 °C for 20 min, and was washed with PBS buffer for future use.

### Property of cage scaffold system

Field emission scanning electron microscope (FE-SEM, JEOL JSM-7500F, Tokyo, Japan) was used to observe the micromorphology of the cage scaffold systems. The samples were coated with Pt prior to SEM observation. Fourier transform infrared (FTIR, NICOLET 6700, Waltham, Massachusetts, America) was utilized to investigate the structure of Biogel and PLA cage system. The samples were prepared by the potassium bromide disc method and scanned for absorbance of 4000 cm^− 1^ to 500 cm^− 1^.

Unconfined compression tests were performed on PLA cage, Biogel and Biogel loaded cage with Micro-fatigue Tester (E3000LT, Instron, UK). The sample was subjected to a stress relaxation test to obtain the equilibrium unconfined compressive strength. Three specimens of each scaffold type were loaded with a force of 1kN until the load cell platen encountered the sample. After equilibrium was achieved, stress relaxation tests were conducted with a compressive deformation of 0.5 mm/min to 10%. Samples were then released to reach equilibrium of displacement (1200s).

### Release pattern of BMP-2 from cage system

The cage+Biogel+BMP-2 composites were placed into the top compartments of a transwell system (SPL Life Sciences, Korea). The release medium (HBSS) in the lower compartment was firstly refreshed from 3 h after beginning and daily refreshed to the 14th day. The released ratio was obtained via the measurement of the concentration of BMP-2 (2.5 μg/ml) in the release medium by ELISA. The measurements were conducted in duplicate and the data were presented as a cumulative release percentage of the total input.

### Cell viability assay

The biocompatibility and potential toxic risk of the cage+Biogel+BMP-2 composites were also evaluated. The cages were sterilized under UV light for 2 h and washed with DPBS three times, and BMP-2-loaded Biogel was injected into the cages. Separately, mouse mesenchymal stem cells (1 × 10^3^/well) were seeded into 24-well plates and incubated in 0.5 ml MEM alpha medium (Gibco, Life Technologies). The cells were settled overnight and the wells were randomly assigned to control group, BMP-2 (250 ng/ml) group, cage group, cage+Biogel group, cage+Biogel+BMP-2(250 ng/ml) group. The cage group was deemed as negative control group and the cage+Biogel+BMP-2 group was treated as a positive control group. The cell viability was measured with ELISA with the Cellrix® viability assay kit (Medifab Co., Ltd., Korea). Following the instruction, the kit and cells were incubated for 30 min and the absorption at 450 nm was measured. The experiments were duplicated conducted.

### Alkaline phosphatase (ALP) assay and staining

ALP activity is an early marker of osteogenic differentiation and is used to detect the bioactivity of the released BMP-2. The cells were washed twice with DPBS and lysed with 0.2% Triton X-100. After centrifuged at 13000 RPM for 3 min, 20 μl supernatant of the cell lysate was mixed with 160 μl of ALP assay buffer (Sigma) and 20 μl of p-nitrophenylphosphate and was incubated for 30 min at room temperature. The absorbance at 405 nm was measured spectrophotometrically an ELISA machine.

For ALP staining, the cells were washed with DPBS for twice. The fast-blue RR salt (Sigma-Aldrich, Brondby, Denmark) contained 0.25% naphthol AS-MX phosphate alkaline solution was added to each well and were incubated for 30 min at room temperature. Then, the cells were observed by using optical microscopy. The groups were same as the ones in the cell viability assay.

### Real-time PCR

Gene expression was evaluated by real-time PCR using specific primer sets for the selected genes, including ALP, Runx-2, OCN, and BSP (Table 1). Total RNA was isolated on day 3, 7, and 14 with a RNeasy mini kit (Qiagen, USA). Reverse transcription was conducted using the High Capacity cDNA Reverse Transcription Kit (Intron Biotechnology, Korea) following its instructions. After cDNA synthesis, real-time PCR (LightCycler® instrument, Roche) was performed with SYBR Green PCR Mastermix. GAPDH was used as an endogenous control. The groups were same as the ones in the cell viability assay.

**Table 1:** Gene specific primer for RT-PCR

Gene	Forward primer	Reverse primer
ALP	ACCATTCCCACGTCTTCACATTT	AGACATTCTCTCGTTCACCGCC
RUNX-2	ATTTCTCACCTCCTCAGCCC	CAACAGCCACAAGTTAGCGA
BSP	CGAATACACGGGCGTCAATG	GTAGCTGTACTCATCTTCATAGGC
OCN	GGCGCTACCTGTATCAATGG	TCAGCCAACTCGTCACAGTC
GAPDH	CCTGTTCGACAGTCAGCCG	CGACCAAATCCGTTGACTCC

### In vivo experiments

#### Rat calvarial defects

The procedures that involved the use of animals for the rat calvarial defect experiment were approved by the international animal care and use committee (SSBMC IACUC No. 2016–0044). Forty-one 8-week-old male Sprague-Dawley (SD) rats (200–220 g) were used in this animal experiment. All animals were kept in specific-pathogen-free housing, provided with abundant food and water and housed under controlled temperature, humidity with a 12:12 dark/light cycle.

The experiments were performed after a one-week stabilization period. The rats were randomly allocated to the following groups: group 1, Cage; group 2, Cage+Biogel; group 3, Cage+BMP-2; and group 4, Cage+Biogel+BMP-2.

**Fig. 1:**
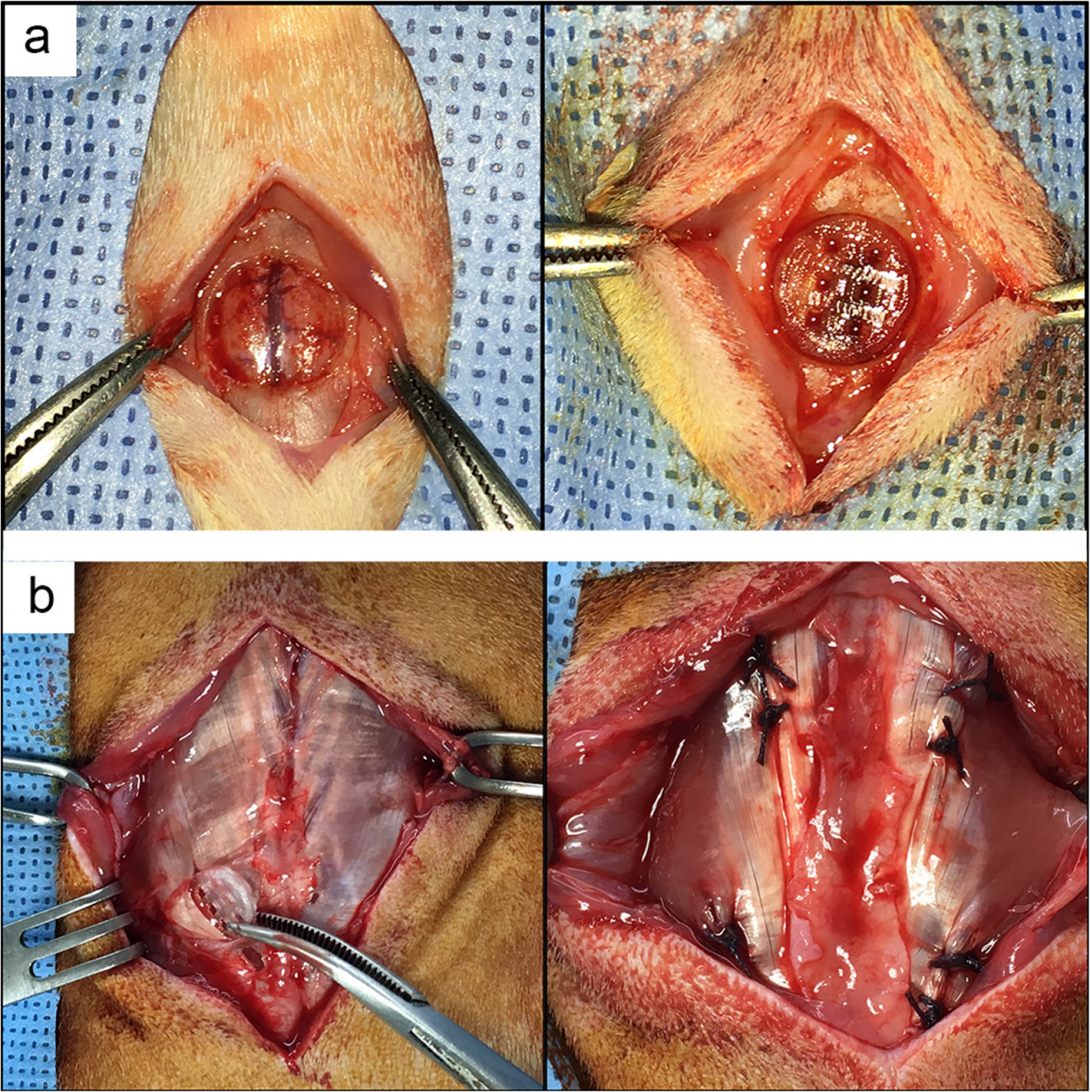
Images of animal experiments. **a** Images of rat calvaria defect. After a round defect with 8 mm diameter defect was made on a rat calvaria, the scaffold was placed in the defect. **b** Images of ectopic ossification model. Four pouches with 10 mm long and 10 mm deep were made in both sides of the back muscle of each rat. After the scaffolds were implanted into the pouches, the incisions were sutured layer by layer

The animals were anesthetized by the intraperitoneal injection of 20 mg/kg Zoletil mixed with 10 mg/kg xylazine, and the surgical procedures were performed in semi-sterile conditions. The surgical site was shaven and sterilized by the application of povidone iodine solution. A longitudinal incision was made over the scalp and the periosteum was dissected. An 8 mm calvarial defect was drilled by using a commercially available trephine bur (Ø 8 mm). The defect was irrigated with normal saline to minimize the thermal injury during the drilling procedure. In additionally, the intactness of the dura mater was carefully preserved to avoid hemorrhage of the sagittal sinus and impairment of bone healing. After the defect was made, the implants of each group were placed into the defect and the opened scalp was sutured. The animals were injected 100 mg/kg cefazolin for 2 days after operation (Fig. 1a).

All animals were sacrificed under deep anesthesia in a CO_2_ chamber in the 8th week after implantation. The implants were carefully harvested with the preservation of peripheral parietal, interparietal, and frontal. All samples were immediately fixed in 10% formalin for micro-CT evaluation and histological assessments.

#### Rat ectopic ossification model

Procedures that involved the use of animals were approved by the international animal care and use committee (SSBMC IACUC No. 2016–0057). Twelve-week-old male SD rats (375–400 g) were used for this animal experiment. Monitoring, stabilizing, and feeding of the rats were same as previously described. The rats were randomly allocated to the following groups: group 1, Cage; group 2, Cage+Biogel; group 3, Cage+BMP-2; and group 4, Cage+Biogel+BMP-2.

The animals were anesthetized by the previously described procedure. The fur on their back was shaved and the exposed skin was sterilized with povidone iodine solution. A 7 cm longitudinal skin incision was made and the iliocostal muscle was exposed with finger and gauze. Four 10 mm long and 10 mm deep pouches were made with a scalpel and hemostats in both sides of the muscle. The pouches on the same side were separated by approximately 15 mm intervals to avoid interference. After the scaffolds were implanted, the muscle layer was sutured using Vicryl and the skin was sutured using Nylon (Fig. 1b).

All animals were euthanized on the 6th week after operation with previously described procedure. The iliocostal muscle from each side was harvested and fixed in 10% formalin immediately for microcomputed tomography and histological assessments.

### Microcomputed tomography (micro CT)

#### Calvarial samples

Tomography projections of the samples were acquired by using a Skyscan 1172 micro-CT scanner (Bruker, Belgium). The samples were scanned with the following setting: pixel size, 9.85 μm; 0.5 mm Al filter; energy, 59 kV; current, 169 μA; rotation step, 0.4°. The cross-sectional images were reconstructed using the NRecon package (Bruker, Belgium) and analyzed with the CT Analyzer software (CT-An, Bruker, Belgium). The threshold values of the newly formed bone were set following the value of host bone. Trabecular bone thickness (Tb.Th), bone volume (BV), and percentage bone volume (BV/TV) were calculated within 8-mm diameter regions of interest (ROI), which were generated based on the defect site.

#### Muscle samples

The muscle samples were scanned by using the same device as for the calvarial samples, with the following parameters: pixel size, 9.85 μm; energy, 49 kV; current, 200 μA; rotation step, 0.7°. The images were processed as previously described.

### Histological assessment

The samples were fixed in 10% formalin and sequentially dehydrated in 80 to 100% ethyl alcohol, infiltrated, and embedded in Technovit 7200 resin (EXAKT, Germany). The resin was solidified with a polymerization system (EXAKT, Germany), the hardened resin blocks were sectioned by using a cutting system (EXAKT, Germany) to 200 μm thick slices, and the slices were ground to a thickness of 50 μm by using a grinding system (EXAKT, Germany). The ground slices were stained with hematoxylin and eosin and the stained bone formations in the scaffolds were observed by using a microscope and a digital camera.

### Statistics

ANOVA method was used to compare between groups with Bonferroni post hoc analysis. (SPSS, IBM) *P* value less than 0.05 was deemed as statistically significant.

## Results

### Characterization of cage for drug delivery

**Fig. 2:**
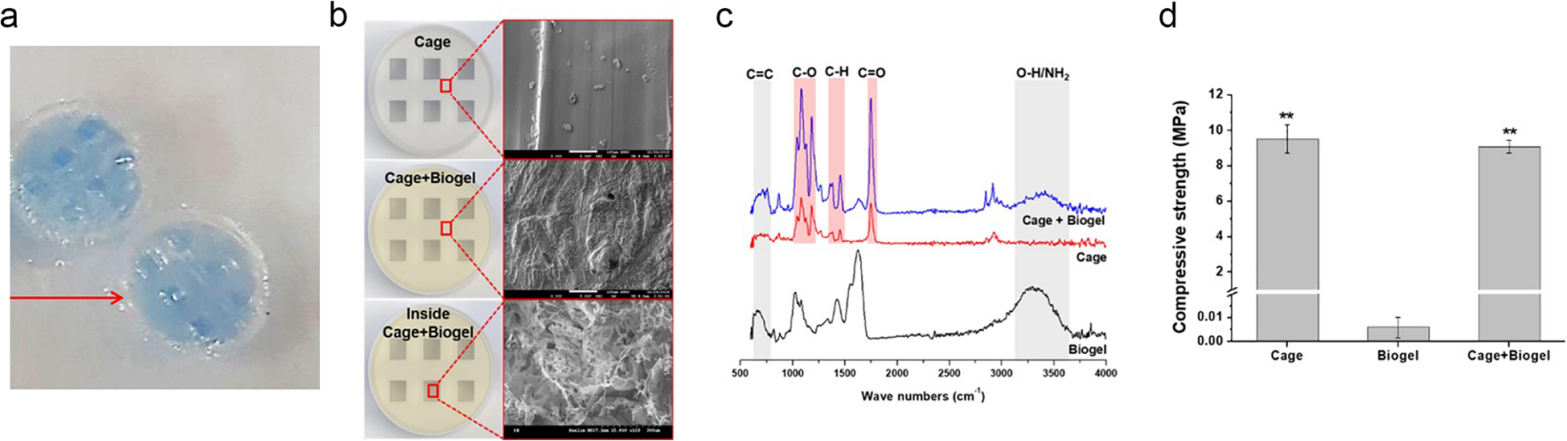
Characteristics of the implants. **a** Confirmation of hollow cavity of the cage. The hollow cavity of the cage was confirmed by the injection of blue ink mixed Biogel. The maximum loading capacity of the PLA cage was 35 μL. **b** SEM images of the scaffold. The surface and inner cavity of the cage were well covered with porous structured Biogel. **c** FT-IR spectroscopy result of scaffold. The spectroscopy indicated the Biogel was composed of alginate and gelatin. **d** The unconfined compression test result. The cage and cage+Biogel groups showed significantly higher compressive modulus than the Biogel group. No significant difference was found between cage and cage+Biogel groups

The hollow cavity of the cage was confirmed by the injection of blue ink mixed Biogel and the maximum loading capacity of the PLA cage was 35 μL. (Fig. 2a) The structural morphology of cage scaffold infilled with Biogel was characterized by scanning electron microscopy (SEM) after sputter coating the samples with Pt (Fig. 2b). The surface and inner cavity of the cage were well covered with porous structured Biogel. FT-IR spectroscopy results showed the characteristic absorption bands of its polysaccharide structure [15], which indicated the Biogel was composed of alginate and gelatin.(Fig. 2c) The hydroxyl (−OH), amino (−NH2) groups of gelatin stretching bands also appeared in the FT-IR spectrogram.

In the unconfined compression test, the cage and cage+Biogel groups showed significantly higher compressive modulus than the Biogel group and no significant difference was found between cage and cage+Biogel groups. Therefore, by loading Biogel into the PLA scaffolds, the advantages of Biogel are preserved, while the mechanical performance are warranted by the PLA scaffolds (Fig. 2d).

### Release profile of BMP-2

**Fig. 3:**
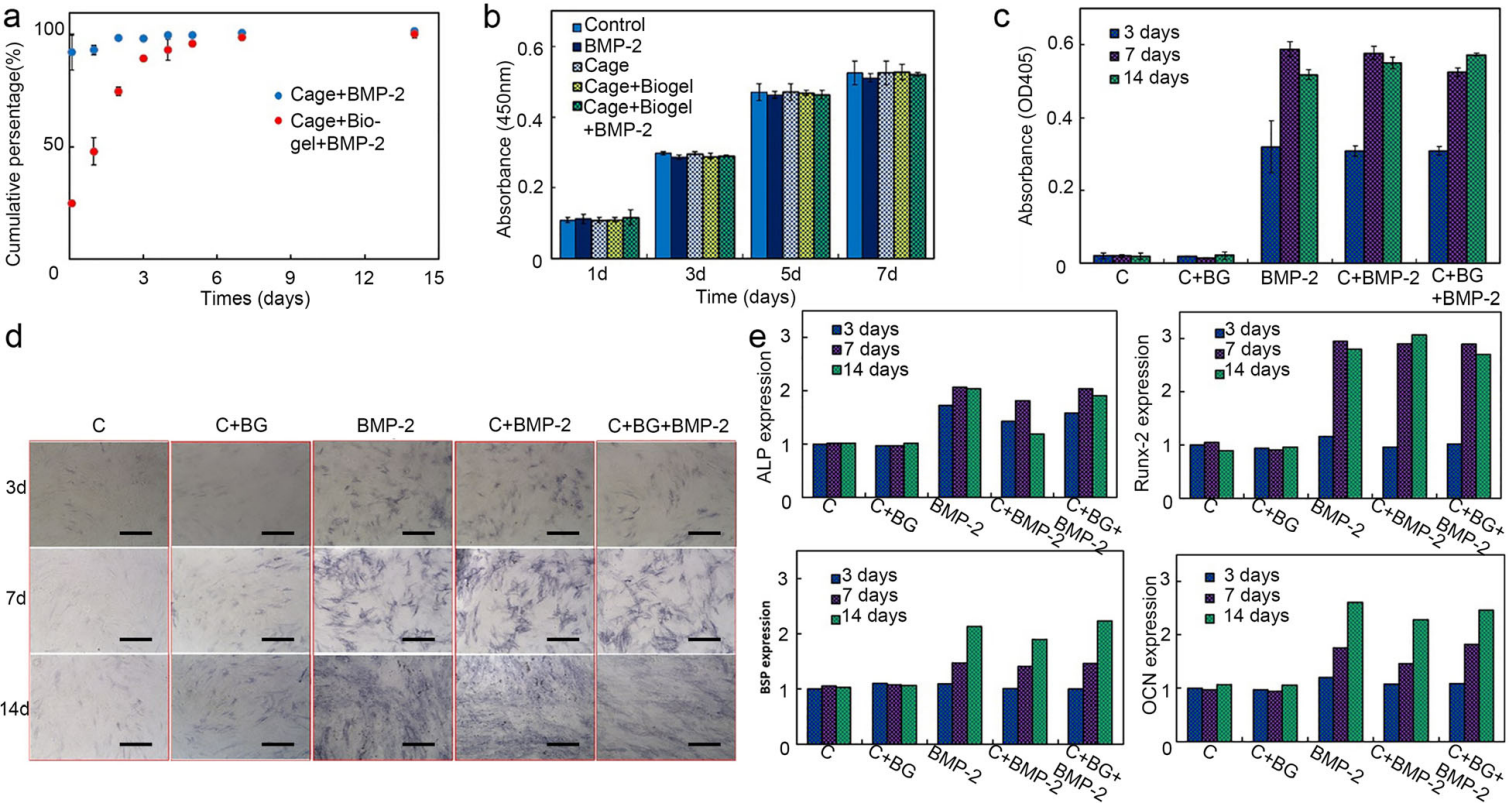
Assessment of osteoinductivity of BMP-2 released from PLA/Biogel scaffold. **a** Releasing profile of BMP-2 from cage and cage/Biogel. About 90% of BMP-2 was released in the first 3 h from the PLA cage, while the 85% of the BMP-2 was released in 4 days in the PLA cage/Biogel group. (*n* = 2) **b** Cell viability assessment. The PLA or Biogel showed no significant cytotoxic effects on mMSCs. (*n* = 2) **c** ALP assay result. The results showed the ALP activity of PLA cage/Biogel/BMP-2 group was comparable with BMP-2 group. (*n* = 2) d, ALP staining result. The PLA cage/Biogel/BMP-2 group showed comparable ALP staining with BMP-2 and PLA cage/Biogel group. Scale bar: 250 μm. e, RT-PCR results. The PLA cage/Biogel/BMP-2 group showed comparable transcription levels of ALP, Runx-2, BSP and OCN. (*n* = 2)

In the groups that the BMP-2 was directly injected into the PLA cage, approximately 90% of BMP-2 was released in the first 3 h, and the remained BMP-2 was under the lower detecting limit at the following timepoints. On the other hand, the releasing ratio of BMP-2 became much slower while it was mixed with Biogel and 85% of the BMP-2 was released in 4 days. Also, we observed a biphasic profile of BMP-2 releasing in cage+Biogel+BMP-2 group, after an initial fast release phase in the first 4 days, comes a sustained slow-release phase for the rest times (Fig. 3a).

### Cell proliferation assay

The patterns of cell proliferation in the cage+Biogel and cage+Biogel+BMP-2 were similar with that of the control group. These results showed that the PLA cage and Biogel did not present cytotoxic effects on mMSCs proliferation and had good biocompatibility (Fig. 3b).

### Osteoinductivity effect of BMP-2 released from the cage+biogel composites

The cage and cage+Biogel groups barely had any increase in ALP assay within the 2 weeks, but the BMP-2, cage+BMP-2 and cage+Biogel+BMP-2 group showed obvious increase from the 3rd day to the 14th day. The BMP-2 group had similar pattern with cage+BMP-2 group and both groups had the highest ALP activity on the 7th day. The cage+Biogel+BMP-2 group showed a gradually increased ALP activity and had the highest level on the 14th day. The result indicated the osteoinductivity of BMP-2 last for at least 14 days in vitro (Fig. 3c) Similar pattern was observed in the ALP staining result. The cage and cage+Biogel groups showed mere staining from the 3rd to 14th day, while the groups with BMP-2 showed a gradually increased staining from the 3rd to 14th day (Fig. 3d).

The expression levels of the bone-related genes had significant increase in the groups with BMP-2, compared with the cage and cage+Biogel groups in the 14 days. No significant difference was observed between the BMP-2, cage+BMP-2 and cage+Biogel+BMP-2 groups (Fig. 3e). The in vitro results consistently showed the BMP-2 released from cage+Biogel composites had comparable osteoinductivity as the BMP-2 itself.

### New bone formation in rat calvaria defect

The cage+BMP-2 and cage+Biogel+BMP-2 showed approximately 8-fold higher BV/TV than the cage and cage+Biogel groups. (Table 2) Also, the cage+BMP-2 and cage+Biogel+BMP-2 groups showed significantly more Tb.N and significantly narrower Tb.Sp than the other two groups. Between the two BMP-2-loaded groups, the cage+Biogel+BMP-2 group had the higher BV/TV and significantly lower Tb.Pf and SMI than the cage+BMP-2 group. (Table 2) Briefly, the results indicated the BMP-2 released from the cage+Biogel carrier induced significantly better bone regeneration than the control groups. Also, between the groups with BMP-2, the group with Biogel showed more bone formation with more plate shape bone.

**Table 2:** The quantitative analysis of micro-CT for the BMP-2 release profile on rat critical-sized calvarial defect model. Data was presented as mean ± SD. Cage *N* = 9, Cage+Biogel *N* = 11, Cage +BMP-2 *N* = 12, Cage+Biogel+BMP-2 *N* = 9

	Cage (*N* = 9)	Cage+Biogel (*N* = 11)	Cage+BMP-2 (*N* = 12)	Cage+Biogel+BMP-2 (*N* = 9)
BV/TV	3.224 ± 2.503	2.945 ± 1.276	20.886 ± 7.67^a,b^	23.414 ± 4.518^a,b^
BS.BV	20.145 ± 2.36	19.631 ± 4.044	17.049 ± 2.918	16.73 ± 1.799
Tb.Th	0.195 ± 0.022	0.178 ± 0.034	0.194 ± 0.031	0.164 ± 0.012
Tb.Sp	1.74 ± 0.313	2.025 ± 0.282	0.701 ± 0.336^a,b^	0.726 ± 0.168^a,b^
Tb.N	0.166 ± 0.129	0.169 ± 0.077	1.104 ± 0.437^a,b^	1.434 ± 0.304^a,b^
Tb.Pf	5.023 ± 9.724	−6.693 ± 8.014	5.34 ± 15.973	−17.572 ± 4.28^a,c^
SMI	2.026 ± 2.021	−0.424 ± 1.697	1.935 ± 3.825	−3.962 ± 1.533^a,b,c^
DA	2 ± 0.473	2.275 ± 0.482	1.802 ± 0.207^b^	−1.718 ± 0.137^b^

^a^ compare with Cage group, *p* < 0.05

^b^ compare with Cage+Biogel group, *p* < 0.05

^c^ compare with Cage+BMP group, *p* < 0.05

**Fig. 4:**
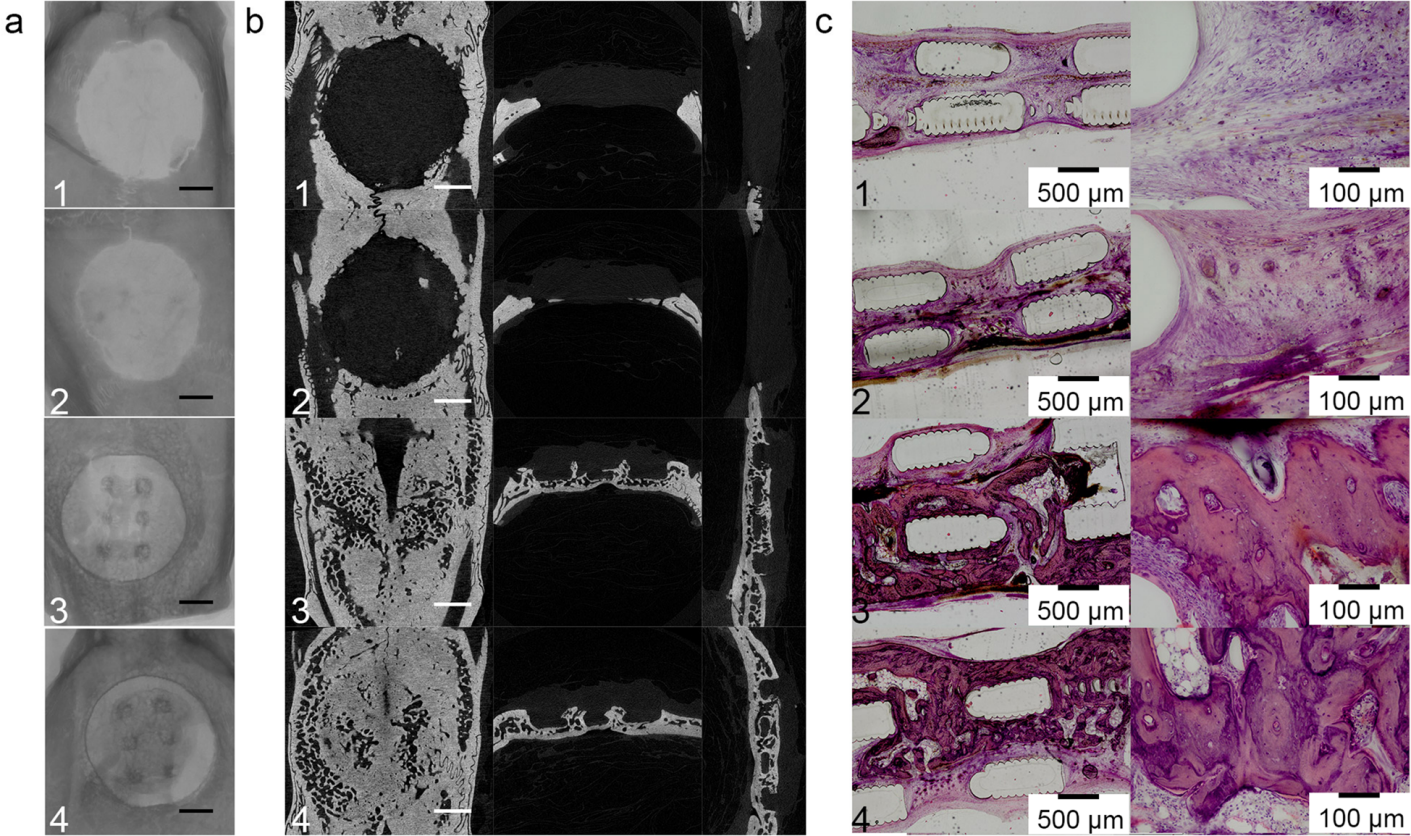
In vivo result of rat calvaria. **a** PLA cage/BMP-2 group and PLA cage/Biogel/BMP-2 group both showed significant bone regeneration. Scale bar: 2 mm. **b** Cross-sectional images of rat calvaria. Both the groups with BMP-2 showed bone regeneration that bridged both edges of defect. Scale bar: 2 mm. **c** Histology sections of rat calvaria. The histology sections confirmed the results of micro-CT. Groups: 1, PLA cage group, *N* = 9; 2, PLA cage/Biogel group, *N* = 11; 3, PLA cage/BMP-2 group, *N* = 12; 4, PLA cage/Biogel/BMP-2 group, *N* = 9

As presented in the representative cross-sectional images, the cage and cage+Biogel groups showed very little bone formation in the defect 8 weeks after surgery. In contrast, the cage+BMP-2 and cage+Biogel+BMP-2 group had large amounts of new bone formation that bridged the defect, and the bone was also well distributed and interconnected (Fig. 4a). The histology sections reconfirmed the results, the cage+BMP-2 and cage+Biogel+BMP-2 showed new bone that filled a vast surface and bridged the defect area (Fig. 4b).

### Ectopic bone formation in muscle tissue

**Fig. 5:**
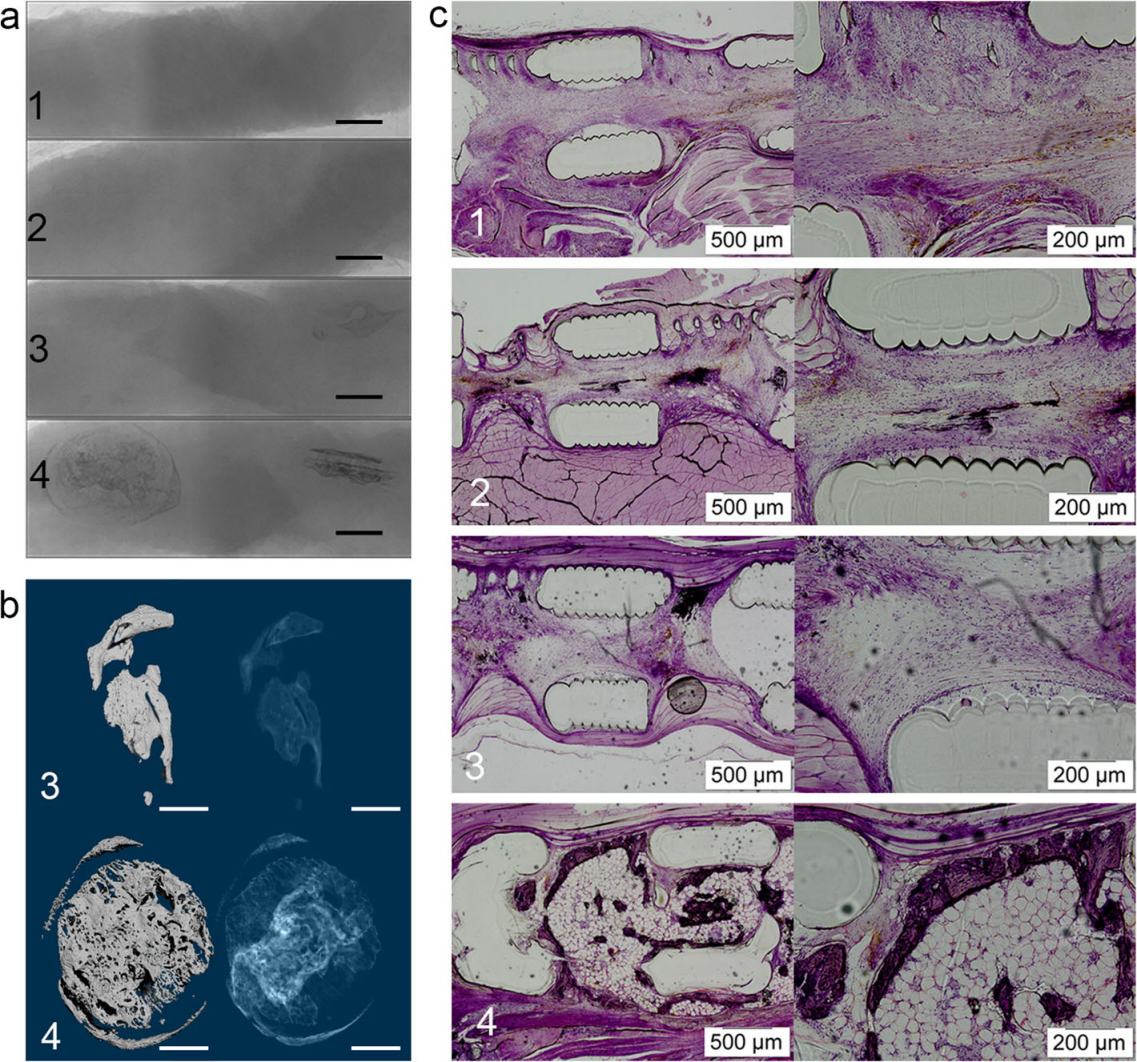
In vivo result of ectopic ossification. **a** The groups without BMP-2 showed no bone formation in the muscle tissue (Group 1 & 2). The PLA cage/BMP-2 and PLA cage/Biogel/BMP-2 groups showed ectopic ossification. The PLA cage/Biogel/BMP-2 group had regular scaffold shaped bone at the implanted sites inside the muscle while the ectopic ossification of PLA cage/BMP-2 group showed irregular shape. Scale bar = 3 mm. **b** Reconstructed 3D images of PLA cage/BMP-2 and PLA cage/Biogel/BMP-2. The PLA cage/Biogel/BMP-2 showed obviously more regular shape bone formation. Scale bar = 2 mm. c, Histology sections of samples. Barely any bone showed in PLA cage group, PLA cage/Biogel group or PLA cage/BMP-2 group. The PLA cage/Biogel/BMP-2 group showed evident bone regeneration inside the scaffold. (Groups: 1, PLA cage group, *N* = 12, PLA cage/Biogel group, *N* = 12; 3, PLA cage/BMP-2 group, *N* = 12; 4, PLA cage/Biogel/BMP-2 group, *N* = 12)

From the raw images of the samples, we noticed the PLA cages were radio translucent and no bone was formed in the cage and cage+Biogel group. The cage+BMP-2 and cage+Biogel+BMP-2 groups showed bone formation inside the muscle tissues, while the shapes of the two groups showed significant differences (Fig. 5a) The new bone in the cage+Biogel+BMP-2 group formed inside and adhesive the cage and formed following the shape of the cage. But in the groups without Biogel, the bone formed irregularly and outside the cage (Fig. 5b) The micro-CT analysis of the Cage and Cage+Biogel was not applicable and therefore, only the BV of the cage+BMP-2 and cage+Biogel+BMP-2 group was compared. The cage+Biogel+BMP-2 had approximately 2.2-fold of new bone formation than the Cage+BMP-2 group did, with statistical significance (Table 3). As the 3D reconstructed images of the two groups presented, the cage+Biogel+BMP-2 group showed a centripetal shape at the center and periphery of the cages. While the bone in cage+BMP-2 group had an irregular form that cannot present the shape of the cage. In the representative histological sections, only the cage+Biogel+BMP-2 group had bone formation inside the cage (Fig. 5c). Generally, the results indicated the Biogel released the BMP-2 following a controllable pattern and induced bone formation adhesive to the cage and avoided unnecessary osteogenesis outside the cage.

**Table 3:** The volume of ectopically formed bone. Data was presented as mean ± SD

Parameter	Cage+BMP-2 (*n* = 12)	Cage+Biogel+BMP-2 (*n* = 12)
BV	2.663 ± 3.116	5.926 ± 2.539*

* *p* < 0.05

## Discussion

The 3D printing technique has been widely applied to treat bone defects for its outstanding customizability that allows for on-demand patient-specific grafts with precise pore size, geometry, and inter-connectivity of the scaffold [16]. Previously we fabricated a carrier system for BMP-2 with PLA scaffold and Hydrogel-based Biogel and proved it has great biocompatibility and potential as an effective bone graft substitute [17]. In this study, we furtherly investigated its effect as the carrier for BMP-2 on inducing bone regeneration following the designed shape.

The PLA cage was designed to provide the mechanical strength and the compressive modulus of our cage/Biogel scaffold was significantly increased higher than Biogel alone. The compressive strength of the scaffold was about 9.3 MPa, which is similar to that of cancellous bone tissue [18] and is much higher than that of other widely used polymeric scaffolds that were made of PLGA or PCL [19, 20]. As presented, BMP-2 loaded in the Biogel/PLA scaffold released followed a biphasic pattern that has been proved to promote better bone formation than a mere burst release [21–23].

In the in vitro part, we presumed the BMP-2 group should have the highest osteoinductivity because the BMP-2 group was fulfilled with freshly made BMP-2 solution but the cage/Biogel/BMP-2 group were only changed with the basic medium. However, the results showed the cage/Biogel/BMP-2 group had comparable ALP activity, ALP stain and expression levels of osteogenesis-related genes with the BMP-2 group did at all 3 time points. The results indicated the bioactivity of the BMP-2 in the cage/Biogel/BMP-2 group maintained at a comparable level with fresh BMP-2 for at least 14 days and induced comparable osteoblastic differentiation.

The in vivo results indicated the scaffolds with BMP-2 successfully induced better bone regeneration than the other groups without BMP-2 in rat calvaria defect. In the further analysis, we observed only a trend of higher BV/TV in cage+Biogel+BMP-2 group than in the cage+BPM-2 group without statistical significance. It was somehow out of our expectation because adding the Biogel has been proved to be able to improve osteogenesis in the previous study [17]. The possible reason for this difference could be the shape of the bone defect. In the calvaria defect, the cavity formed by the dura and the host bone helps the PLA cage to retain the BMP-2 in the defect for a sufficient time to induce bone regeneration. The Tb.Pf and SMI results indicated the cage/Biogel/BMP-2 group induced more plate-shaped bone that had significantly higher continuity and more enclosed cavities. Therefore, adding Biogel in the PLA scaffold could induce bone regeneration with a more rigid structure.

In the ectopic ossification model, the superior effect of sustainable and controllable releasing of bioactive BMP-2 became evident and could be easily distinguished from other groups. Only the cage/Biogel/BMP-2 group formed regular-shaped bone around the scaffold in the defect site, while the bone in the cage/BMP-2 group formed outside the original implanted site, and no bone formed in the groups without BMP-2. EO or heterotopic ossification is one of the complications of BMP-2 which is frequently reported to cause a series of complications such as overt dysfunction, soft-tissue loss, joint contractures, and chronic pain [24–26]. Therefore, an optimal delivery system should release the BMP-2 under a controllable ratio and guide the shape of the forming bone [27]. Our results indicated the Biogel successfully controlled the releasing of BMP-2 from the scaffold and avoided EO in muscle tissues.

Some limitations remained in this study. Only one kind of Biogel was used at the current stage, since tuning the composition of Biogel can control the releasing pattern of BMP-2 and affect the bone formation, the Biogels with a different releasing pattern of the BMP-2 should be furtherly investigated to optimize the effect. Also, the calvaria defect had no mechanical compress at the defect sites, further studies should be performed on the load-bearing bone defects of large animals for a better understanding of the effect of a carrier under the clinical situation. Also, the process of manufacturing the scaffold should be furtherly improved to minimize the protein activity loss and the risk of contamination.

We noticed the original site of Biogel had been replaced by the newly formed bone inside the scaffold, but the PLA material still possessed its space and did not show apparent degradation. The long degradation period of PLA has the advantage of offering a certain degree of mechanical strength and protecting the newly regenerated tissues, but it also delays the regeneration progress because the space cannot be replaced by the bone tissue [28]. Therefore, further investigation should focus on the balance between the mechanical strength and biodegradation ratio for optimized bone regeneration.

## Conclusions

The PLA/Biogel scaffold was proved to be a biocompatible carrier system of the BMP-2, which could sustainably release the BMP-2 and induce bone formation following the originally designed shape. Therefore, the carrier system possessed great potential for clinical use for inducing bone regeneration and reducing the risk of EO.

## Supplementary Information

**Additional file 1.**

## Data Availability

The datasets used and/or analysed during the current study are available from the corresponding author on reasonable request.
