# Supplementary figures and images for: Three-dimensional printed polylactic acid scaffold integrated with BMP-2 laden hydrogel for precise bone regeneration
Source: Biomater Res. 2021 Oct 27;25:35. doi: 10.1186/s40824-021-00233-7 (PMC8554986; doi:10.1186/s40824-021-00233-7)

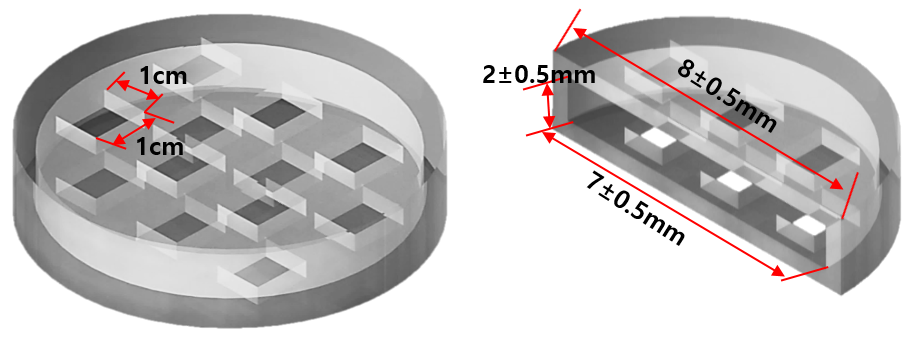

Supplement: Supplementary file 1 — Additional file 1. [file 40824_2021_233_MOESM1_ESM.tif]
